# Factors explaining variation in self-esteem among persons with type 1 diabetes and elevated HbA1c

**DOI:** 10.1371/journal.pone.0201006

**Published:** 2018-08-10

**Authors:** Jannike Mohn, Jannicke Igland, Vibeke Zoffmann, Mark Peyrot, Marit Graue

**Affiliations:** 1 Centre for Evidence-Based Practice, Western Norway University of Applied Sciences, Bergen, Norway; 2 Department of Global Public Health and Primary Care, University of Bergen, Bergen, Norway; 3 Section of Endocrinology, Department of Medicine, Haukeland University Hospital, Bergen, Norway; 4 Research Unit Women’s and Children’s Health, The Juliane Marie Centre, Copenhagen University Hospital, Rigshospitalet, Copenhagen, Denmark; 5 Department of Sociology, Loyola University Maryland, Baltimore, Maryland, United States of America; University of Colorado Denver School of Medicine, UNITED STATES

## Abstract

**Objectives:**

To investigate associations between perceived autonomy support from health-care professionals, autonomy-driven motivation, diabetes self-perceived competence and self-esteem in adults (age 18–55 yrs) with suboptimally regulated type 1 diabetes mellitus (T1DM) with at least one HbA1c≥8.0% (≥64 mmol/mol) during the past year, and whether these factors could predict decrease in self-esteem over time.

**Methods:**

A cross-sectional population-based survey was performed, and 9 months follow-up data were collected. Data collection comprised clinical and socio-demographic variables, blood sampling (HbA_1c)_ and self-report questionnaires; the Health Care Climate Questionnaire (HCCQ), Treatment Self-Regulation Questionnaire (TSRQ), the Perceived Competence in Diabetes Scale (PCDS), and the Rosenberg Self-esteem Scale (RSES). We fitted block-wise linear regression models to assess associations between RSES and variables of interest (HCCQ, TSRQ, PCDS, HbA_1c_, clinical and socio-demographic variables) and linear regression models to assess predictors of change over time.

**Findings:**

In this study sample, aged 36.7 (±10.7) mean HbA_1c_ 9.3% (±1.1), 31.5% had long-term complications and 42.7% had experienced severe hypoglycemia within the previous 12 months. In the final regression model the association between PCDS and RSES was strongly significant (B = 1.99, *p*<0.001) and the associations between HCCQ, TSRQ and RSES were reduced to non-significance. All predictor variables combined explained 42% of the variability of RSES (adjusted R^2^ = 0.423) with PCDS contributing 18% to explained variance (R-square change = 0.184, *p*<0.001). The strongest predictors of change in RSES over time were long-term complications (B = 2.76, *p<0*.*001*), specifically foot-related problems, and being female (B = -2.16, *p* = 0.002).

**Conclusions:**

Perceived autonomy support, autonomy-driven motivation and diabetes self-perceived competence play a significant role in explaining self-esteem among adults with suboptimally regulated T1DM. Healthcare professionals should acknowledge self-esteem as a valuable factor in understanding the multifaceted health choices people with T1DM make.

**Trial registration:**

Clinical Trials.gov with identification number NCT 01317459.

## Introduction

Diabetes mellitus (DM) is a complex chronic condition [1] leaving the individual with daily demanding treatment-related choices [2]. To understand the health choices people with chronic illness make, Luyckx and colleagues explored the illness self-concept: ‘the extent to which chronic illness becomes integrated in the self’ [3], and the degree to which type 1 diabetes (T1DM) intrudes upon one’s self [4]. An individual’s self-concept can be understood as a collection of cognitive or descriptive beliefs about one’s self from which he or she derives a sense of self-worth or *self-esteem* [5, 6]. Luyckx pointed out that the extent to which chronic illness becomes fully integrated in the self is significantly related to self-esteem, defined as ‘the degree to which an individual has a favorable or unfavorable opinion of himself and finds himself worthy or unworthy’ [7].

In a 10-year follow-up study, researchers found that self-esteem was lower among young adults with diabetes than among those without a chronic illness [8]. In a study among 478 emerging adults with T1DM women reported lower self-esteem than men [9]. Compared to healthy individuals, women with diabetes reported lower self-esteem, while men with diabetes reported higher levels of self-esteem. In a Danish study, as well as in a study from the US., participants who reported high levels of diabetes-related emotional distress were found to score low on self-esteem, a fundamental concern when considering the relatively high rates of diabetes distress among persons with T1DM [10, 11]. The extent to which chronic illness become integrated in the self among persons with diabetes may constitute a barrier to self-management, a matter of vital importance in the diabetes field [12].

There is evidence that problematic diabetes self-management behavior is associated with psychological distress [13, 14] and, in turn, with poor glycaemic control [15, 16]. In order to manage and maintain adequate self-management, motivation has been identified as a key concept [17–19]. According to a general theory of motivation, the Self-Determination Theory (SDT), the extent to which individuals perceive relatedness (feeling understood and cared for by others), autonomy (feeling of being the origin of one’s own behaviour) and competence (feeling effective) enhance ownership and internalization of their behaviour [20, 21]. SDT focuses on both the contextual and motivational factors that facilitate psychological functioning, and postulates that individuals are autonomously motivated if they feel a sense of choice, volition and congruence of their behaviour with their personal values [20]. In light of SDT, we have previously shown that autonomy support from health care professionals (HCPs) was associated with higher self-perceived diabetes competence, which in turn was associated with lower level of diabetes-related emotional distress among adults with T1DM and chronically elevated HbA_1c_ [22]. Moreover, in a randomized controlled trial within the same population [23], we have evaluated the effect of a group-based behavioral intervention among adults with T1DM. The intervention promotes patient autonomy, participation, skills building and intrinsic motivation, and is called Guided Self-Determination’ [24, 25]. The intervention gave significant effects on diabetes distress, autonomy-driven motivation and self-esteem nine months post intervention. Interestingly, the self-esteem level among intervention group participants remained stable, whereas, among control group participants, self-esteem decreased significantly.

The objective of the present study was to broaden our understanding of how relatedness (with laypersons and HCPs), autonomy-driven motivation and self-perceived competence might be associated with self-esteem among adults with suboptimally regulated T1DM, and whether these factors could predict a decrease in self-esteem over time.

## Methods

### Study design and research setting

The data used in the present study are mainly baseline-data from the randomized controlled group-based intervention among adults with suboptimal controlled T1DM (identifier NCT 01317459). In order to assess explained variance in change in self-esteem among control group participants at follow-up, 9 months results of self-esteem were also included. The study took place at a diabetes out-patient clinic at a university hospital in Western Norway, and patients were enrolled from March 2011 to March 2013. The hospital’s catchment population is ethnically homogeneous and stable, and includes both urban and rural populations.

### Recruitment and participants

Persons with T1DM aged 18–55 yrs scheduled to attend consultations (*n* = 561) were assessed for eligibility according to the study’s inclusion/exclusion criteria. Inclusion criteria were: HbA_1c_ ≥8.0% (≥64 mmol/mol) on one or two occasions during the year prior to the study and at least two daily insulin injections or continuous subcutaneous insulin infusion. Exclusion criteria were: severe medical co-morbidity, major psychiatric diagnosis, cognitive deficiency, inadequate reading/speaking skills in Norwegian, pregnancy, visual impairment that prevented reading or substance abuse. Further details on recruitment and participants are available elsewhere [23].

Those persons who neither responded to the postal request nor came for their scheduled appointment at the clinic were classified as non-responders (*n* = 149). Of the remaining eligible population (*n* = 327) another 149 patients actively declined participation (response given verbally when they were at the clinic or by telephone if they were unable to meet for their scheduled appointment), leaving a final study population of 178 consenting participants.

### Assessments

Participants completed a self-report questionnaire consisting of demographic information: age, sex, level of education, employment status and marital/co-habitation status. Participants were also asked to report disease-related information: insulin treatment regimen, diabetes duration, hypoglycaemia episodes and diabetes long-term complications (heart failure, stroke, end-stage renal disease, retinopathy, digestive problems, foot-related problems, lower urinary tract symptoms) [23]. All questions about complications had three possible answers: ‘yes’, ‘no’ and ‘don’t know’. To increase power these variables were combined into ‘any long term complications’, ‘yes’ or ‘no/don’t know’. In addition, all participants reported the number of self-monitored blood glucose measurements (SMBG) completed in the past two weeks in the following categories: ‘no monitoring last 14 days’; ‘less than every week’; ‘less than every day’; ‘1–3 measurements per day’; ‘4–6 measurements per day’ and ‘7 or more measurements per day’. The SMBG frequency was analyzed in the following three categories; ‘less than every day’; ‘1–3 times per day’ and ‘4 or more measurements per day’. All participants had HbA_1c_ assessed in connection with their regularly scheduled visit at the clinic. Samples were analyzed at the university hospital using high-performance liquid chromatography (DCA Vantage/Siemens, DCA 2000 and DCA 2000+/Bayer), assays standardized and calibrated against the IFCC—International federation of Clinical Chemists standards [12]. All the participants completed the following instruments assessing psychosocial functioning:

1. The *Rosenberg Self-Esteem Scale* (RSES) measures one’s overall self-esteem with an equal number of positively (e.g. ‘On the whole, I am satisfied with myself’) and negatively (e.g. ‘I feel I do not have much to be proud of’) worded items [7]. The responses are rated on a four-point Likert scale indicating level of agreement (1–4, ranging from ‘strongly disagree’ to ‘strongly agree’). A total scale score is computed by summing the item responses for the 10 items. Higher scores represent better self-esteem, and scores below 15 suggest low self-esteem. The RSES scale has been demonstrated to have high internal consistency in the Norwegian populations (Cronbach’s α = 0.86) [26]. In the current study, the Cronbach’s alpha coefficient was 0.90.
2. The *Health Care Climate Questionnaire* (HCCQ) assesses patients’ perceptions of the degree to which their health-care providers (HCPs) are supportive of autonomy rather than controlling in consultations [27]. In this study we used the short form containing 6 statements (e.g., ‘I feel that my health-care providers provide me with choices and options’) rated on a seven-point Likert scale indicating level of agreement (1–7, ranging from ‘strongly disagree’ to ‘strongly agree’). Higher scores represent greater perceived support for autonomy by HCPs. The six-item short form has a Cronbach’s alpha coefficient of 0.82 [27]. In the current study, the alpha coefficient was 0.95.
3. The *Treatment Self-Regulation Questionnaire* (TSRQ) assesses the diabetes self-care practices and whether this behavior is controlled (external) or self-motivated (autonomous/internal) [28]. The questionnaire assesses why the person with diabetes has a certain behavior and then provides several preselected possible reasons representing different styles of regulation/motivation. The statements are rated on a seven-point Likert scale indicating level of accuracy (1–7 ranging from ‘not at all true’ to ‘very true’). The questionnaire has two subscales; Autonomous regulation (TSRQ Autonomy, 8 statements) and Controlled regulation (TSRQ Control, 11 statements). Each participant gets a score by averaging responses to each of the items that make up that subscale. There is good evidence of the stability of the TSRQ scale across various health domains demonstrating an acceptable internal consistency of each subscale (most Cronbach’s alpha values >0.73) [28]. The Cronbach’s alphas in the current study were 0.81 for TSRQ Autonomy, 0.87 for TSRQ Control and 0.87 for all the 19 TSRQ statements.
4. The *Perceived Competence for Diabetes Scale* (PCDS) assesses the degree to which persons with diabetes feel they can manage the every-day aspects of diabetes care (29). The PCDS contains 4 statements (e.g., ‘I am able to manage my diabetes’) rated on a seven-point Likert scale indicating level of agreement (1–7 ranging from ‘not at all true’ to ‘very true’); higher scores represent better respondent’s performance. The mean of a person’s responses is used as a summary score. The PCDS has internal consistencies of Cronbach’s alpha ranging between 0.83 and 0.93 [27, 29]. In the current study, the Cronbach’s alpha coefficient was 0.92.

### Statistical analysis

To analyze the difference between men and women we used χ^2^ and Mann-Whitney *U* tests. To explore the association between self-esteem (RSES) at baseline and a set of predictors we used an unadjusted linear regression model for each predictor and a block-wise multiple linear regression model based on theories of human motivation as outlined in SDT. The blocks of variables were entered according to their hypothesized causal ordering [30]: (A) socio-demographic (age, sex, level of education, co-habitation status and employment status) and clinical factors (long term complications, insulin treatment regimen, SMBG, diabetes duration, episodes of severe hypoglycaemia and HbA_1c_), (B) level of perceived autonomy support from HCPs (HCCQ), (C) autonomy-driven motivation (TSRQ Autonomy) and (D) self-reported level of diabetes self-perceived competence (PCDS). Change in self-esteem from baseline to follow-up in the control groups was tested using paired t-test. To assess factors that could predict a drop in self-esteem among control group participants over time, we calculated a change score for RSES as the difference between RSES at 9 month follow-up and RSES at baseline and used the change score as the dependent variable. We first used separate linear regression models for each predictor with adjustment for baseline-value of self-esteem. Then a multivariate linear regression model was used to expand on explained variance in change in self-esteem at 9 months follow-up.

R-squared change was used to assess explanatory power of each block of variables as the incremental contribution to explained variance. Because certain predictor variables (employment status and SMBG) showed different distributions between men and women, and self-esteem also differed between men and women, we performed interaction analyses to see if the association between the predictors and self-esteem differed between genders. No significant gender-interactions were found for any of the predictors, and results are therefore presented as one common model for both genders.

The association between autonomy support and competence was estimated by linear regression model using competence as the outcome and autonomy support as a predictor with adjustment for demographic and clinical variables. The association between autonomy-driven motivation and competence was estimated in the same manner with competence as the outcome and autonomy-driven motivation as the predictor with the same adjustment variables.

All analyses were performed using SPSS version 23.0 for Windows (SPSS Inc.). Data were screened for outliers and missing values. Missing values were handled by pairwise exclusion. The significance level was set to 0.01 to reduce risk of Type I error because of multiple testing.

### Ethics

The Regional Committees for Medical and Health Research Ethics in Norway approved the study (reference number 2010/1325). In addition, the committee gave permission to record age, gender and HbA1c of the non-responders.

## Results

### Participant characteristics

**Fig 1:**
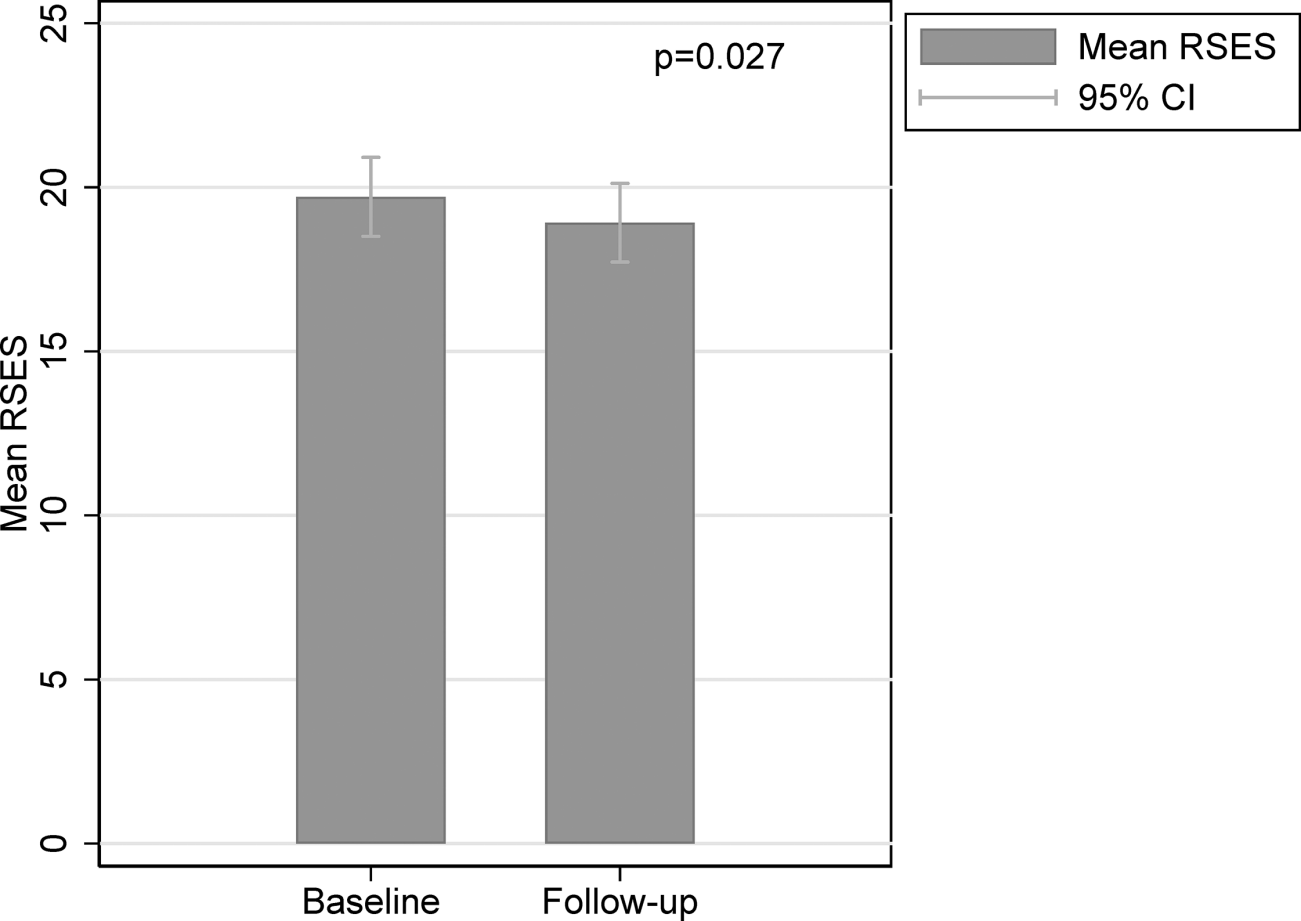
Mean level of self-esteem according to the Rosenberg Self-Esteem Scale (RSES) at baseline and follow-up. P-value from paired t-test comparing follow-up measurement with baseline-measurement.

The mean age of the study sample (*n* = 178) was 36.7 years (±10.7) with no significant differences between genders (Table 1), and about one third of the sample had higher education with no gender differences. Of the total population 13.5% were unemployed, the distribution of employment status significantly differing between men and women (*p* = 0.006). Mean level of HbA_1c_ was 9.3% (±1.1) with a range from 8.0 to 14.3%. Approximately one third of the participants had diabetes-related complications (31.5%), and 42.7% of the participants had experienced severe hypoglycaemia within the previous 12 months. The frequency of self-monitoring of blood glucose (SMBG) tended to differ between genders with 22.4% of women monitoring rarely (‘less than every day’ to ‘no monitoring last 14 days’) versus 36.4% of men, but the difference was not significant (*p* = 0.014). Except for the above-mentioned variables, there were neither significant differences between genders in the demographic or clinical variables, nor in variables measuring psychosocial functioning (self-esteem (RSES); autonomy support (HCCQ); autonomy-driven motivation (TSRQ Autonomy) or diabetes self-perceived competence (PCDS)). The non-responders and those who declined participation (n = 149+149 = 298) did not differ significantly from participants (n = 178) with regard to mean age (34.4 ±11.2 vs 36.7 ±10.7 years; p = 0.032) or HbA1c (9.2 ±1.2% (76 ± 13 mmol/mol) vs 9.3 ±1.1% (78 ± 11 mmol/mol); p = 0.025); however, a significant sex ratio difference was found (male/female: 178/120 versus 67/111; p<0.001). Mean RSES at baseline and at follow-up for the 83 control group participants are displayed in Fig 1. The average reduction in RSES was 0.80, which was not significant (p = 0.03).

**Table 1:** Characteristics of the study participants by gender (N = 178).

		All (n = 178)	Men (n = 67)	Women (n = 111)	p-value*
*Demographic characteristics*					
Age, mean (SD)		36.7 (10.7)	37.7 (10.3)	36.2 (10.9)	0.37
Living alone, n (%)		28 (15.7)	10 (15,2%)	18 (16.1)	0.87
University education, n (%)					
	No University education	114 (64.1)	44 (66.7)	70 (62.5)	
	University education ≤ 4 years	44 (24.7)	16 (24.2)	28 (25.0)	
	University education > 4 years	20 (11.2)	6 (9.1)	14 (12.5)	0.76
Employment status n (%)					
	Working fulltime	120 (67.4)	54 (81.8)	66 (58.9)	
	Working part-time	34 (19.1)	6 (9.1)	28 (25.0)	
	Not working	24 (13.5)	6 (9.1)	18 (16.1)	0.006
*Clinical characteristics*					
Diabetes duration in years, median (range)		19.0 (1–46)	19.8 (1–37)	19.4 (1–46)	0.89
HbA1_c,_ Mmol/mol, mean (SD)		78 (12)	79.3 (12.4)	77.3 (11.7)	
HbA1_c,_ % points, mean (SD)		9.3 (1.1)	9.4 (1.1)	9.2 (1.1)	0.30
Long-term complications total, n (%)		56 (31.5)	15 (22.7)	41 (36.6)	0.05
	Heart failure, n (%)	11 (7.0)	5 (8.9)	6 (5.9)	0.52
	Stroke, n (%)	2 (1.3)	1 (1.9)	1 (1.0)	1.00
	End-stage renal disease, n (%)	7 (4.5)	2 (3.7)	5 (5.0)	1.00
	Retinopathy, n (%)	26 (16.3)	11 (18.6)	15 (14.9)	0.66
	Digestive problems, n (%)	23 (14.7)	3 (5.7)	20 (19.4)	0.03
	Foot-related problems, n (%)	14 (8.8)	5 (8.8)	9 (8.8)	1.00
	Lower urinary tract symptoms	12 (7.8)	2 (3.8)	10 (10.0)	0.22
Insulin pump, n (%)		75 (42.1)	24 (36.4)	51 (45.5)	0.23
Severe hypoglycemia past year, n (%)		76 (42.7)	27 (40.9)	49 (44.5)	0.64
Frequency of Self-Monitoring Blood Glucose, n (%)					
	≥7 times per day	21 (11.8)	4 (6.1)	17 (15.2)	
	4–6 times per day	54 (30.3)	15 (22.7)	39 (34.8)	
	1–3 times per day	54 (30.3)	23 (34.8)	31 (27.7)	
	Less than every day	28 (15.7)	10 (15.2)	18 (16.1)	
	Less than every week	12 (6.7)	9 (13.6)	3 (2.7)	
	No monitoring last 14 days	9 (5.1)	5 (7.6)	4 (3.6)	0.014
*Psychosocial functioning (defined range)*					
RSES^a^, mean (SD)		19.4 (5.7)	20.5 (5.3)	18.8 (5.8)	0.048
HCCQ^b^, mean (SD)		5.0 (1.5)	5.0 (1.4)	5,0 (1.5)	0.966
TSRQ^c^, mean (SD)					
	Autonomy	5.2 (1.1)	4.9 (1.2)	5.3 (1.1)	0.025
	Control	3.3 (1.3)	3.2 (1.3)	3.3 (1.2)	0.753
	Relative Autonomy Index (RAI)	1.9 (1.4)	1.7 (1.4)	2.0 (1.4)	0.130
PCDS^d^, mean (SD)		4.3 (1.5)	4.4 (1.4)	4.3 (1.6)	0.704

^a^RSES, Rosenberg Self-esteem scale

^b^HCCQ, Health Care Climate Questionnaire

^c^TSRQ, Treatment Self-Regulation Questionnaire

^d^PCDS, Perceived Competence in Diabetes Scale

* p-value from Mann Whitney U-test for continuous variables and from Chi-square test for categorical variables

### Predictors of self-esteem in light of SDT

Table 2 displays the results of the block-wise regression analyses for the associations with baseline RSES. In the unadjusted models HCCQ, PCDS and TSRQ Autonomy showed significant positive associations with RSES, indicating that a higher level of the predictors was associated with better self-esteem. The model including only demographic and clinical variables (Step A) accounted for 11.0% of the variation in RSES (adjusted R^2^ = 0.110). When autonomy support (HCCQ) was entered in step B, HCCQ showed a significant positive association with RSES and the explained variance increased significantly from 11% to 15.7%, indicating that 4.7% of the variation in RSES could be explained by HCCQ after taking into account potential confounding demographic and clinical factors. When autonomy-driven motivation (TSRQ Autonomy) was controlled (step C), the regression coefficient for TSRQ Autonomy was positive and significant (B = 1.62, *p*<0.001) while the regression coefficient for HCCQ was reduced from 0.87 to 0.57 and no longer significant at the 0.01 level. TSRQ Autonomy contributed an additional 8.1% to the explained variance in RSES. Finally, when perceived competence (PCDS) was added to the model (Step D) the association between PCDS and RSES was strong and significant (B = 1.99, *p*<0.001). The association between HCCQ and RSES completely disappeared and the regression coefficient for TSRQ Autonomy was reduced from 1.62 to 0.61 and was no longer significant, indicating that both the association between HCCQ and RSES and the association between TSRQ Autonomy and RSES was mediated through PCDS. The regression coefficient between autonomy support (independent variable) and perceived competence (dependent variable) was B = 0.40, *p*<0.001 (not shown in Table 2). The estimate of the regression coefficient between autonomy-driven motivation (independent variable) and perceived competence (dependent variable) was B = 0.61, *p*<0.001 (not shown in Table 2). The adjusted R^2^ (= 0.423) indicates that the variables in the final regression equation (Step D) explained 42% of the variability of self-esteem (RSES) with diabetes competence (PCDS) contributing 19% to the explained variance (R-square change = 0.188, *p*<0.001). In the final model the regression coefficient for ‘Living alone’ was negative and strong, but not significant (B = -2.66, *p* = 0.010).

**Table 2:** Blockwise linear regression analysis of self-esteem among persons with Type 1 DM 18–55 years of age (N = 178).

		Unadjusted analysis ^a^ ^b^				Step A Demographic and clinical				Step B Autonomy Support				Step C Autonomy-driven motivation				Step D Competence			
		B	95% CI		P	B	95% CI		P	B	95% CI		P	B	95% CI		P	B	95% CI		P
Sex (Female)		-1.70	-3.42	0.03	0.05	-1.38	-3.24	0.47	0.14	-1.41	-3.22	0.40	0.13	-2.15	-3.92	-0.39	0.02	-1.66	-3.20	-0.11	0.04
Age		0.06	-0.02	0.14	0.13	0.05	-0.06	0.15	0.36	0.05	-0.05	0.15	0.31	0.01	-0.09	0.11	0.90	0.00	-0.09	0.09	0.997
University education		1.82	0.08	3.55	0.04	1.16	-0.74	3.07	0.23	1.69	-0.20	3.58	0.08	1.65	-0.16	3.45	0.07	1.49	-0.09	3.07	0.06
Living alone		-2.91	-5.19	-0.64	0.01	-2.48	-4.97	0.01	0.05	-2.43	-4.86	0.01	0.05	-2.06	-4.38	0.27	0.08	-2.68	-4.72	-0.63	.010
Employed		1.22	0.06	2.38	0.04	1.00	-0.25	2.26	0.12	1.01	-0.22	2.23	0.11	0.54	-0.65	1.73	0.37	0.62	-0.42	1.66	0.24
Long-term complications		-0.20	-2.01	1.61	0.83	-0.39	-2.34	1.56	0.69	-0.49	-2.40	1.41	0.61	-0.84	-2.66	0.98	0.36	-0.61	-2.21	0.98	0.45
Pump		-0.09	-1.80	1.61	0.92	0.37	-1.48	2.21	0.70	0.63	-1.18	2.44	0.49	0.86	-0.87	2.59	0.33	0.78	-0.32	2.29	0.31
SMBG^c^																					
	1–3 times per day	0.88	-1.12	2.89	0.39	0.33	-1.76	2.42	0.76	0.34	-1.70	2.38	0.74	0.84	-0.12	2.80	0.40	0.11	-1.61	1.84	0.90
	Less than daily	-0.17	-2.23	1.89	0.87	0.23	-1.99	2.45	0.84	0.15	-2.01	2.32	0.89	0.73	-1.35	2.82	0.49	-0.31	-2.15	1.53	0.74
Diabetes duration		0.03	-0.05	0.11	0.49	0.004	-0.10	0.11	0.94	-0.01	-0.11	0.09	0.85	0.01	-0.09	0.11	0.86	-0.02	-0.10	0.07	0.70
Severe hypoglycemia		-0.07	-1.78	1.64	0.94	0.17	-1.60	1.95	0.857	0.52	-1.22	2.27	0.56	0.46	-1.21	2.13	0.59	1.17	-0.30	2.64	0.12
HbA_1c_ (%)		-0.78	-1.55	-0.02	0.04	-0.57	-1.41	0.30	0.20	-0.44	-1.28	0.40	0.31	-0.56	-1.36	0.24	0.17	0.13	-0.60	0.85	0.73
Autonomy support^d^		0.76	0.20	1.32	0.008					0.87	0.29	1.44	0.003	0.57	0.002	1.14	0.05	-0.02	-0.55	0.50	0.93
Autonomy-driven motivation^e^		1.63	0.92	2.34	<0.001									1.62	0.84	2.40	<0.001	0.61	-0.13	1.35	0.11
Diabetes competence^f^		2.08	1.62	2.53	<0.001													1.99	1.43	2.55	< .001
Overall R square						0.110				0.157				0.239				0.423			
Change in R square										0.047			.003	0.081			<0.001	0.184			< .0001

^a^Unstandardized regression coefficients

^b^Each cell in this column represents the coefficients from a bivariate regression analysis

^c^Self-monitored blood glucose measurements, with ≥4 times per day as the reference category

^d^HCCQ, Health Care Climate Questionnaire

^e^TSRQ, Treatment Self-Regulation Questionnaire, Autonomy

^f^PCDS, Perceived Competence in Diabetes Scale

### Predictors of change in self-esteem over time

Table 3 displays the results of the regression analyses performed to predict change in self-esteem among control group participants over time. We found that the strongest predictor of change in RSES at 9 months follow-up in the multivariate model was long-term complications, which was associated with positive change (B = 2.76, *p* <0.001). Because the variable long-term complications was a combination of several complications we also explored how much each of the seven original complication variables contributed to the regression in additional analyses. No complications other than ‘foot-related problems’ showed significant coefficients in separate regression analyses (B = 3.24, *p* 0.006) (not shown in tables). Another significant predictor was being female, which was associated with negatively change in self-esteem (B = -2.16, *p* 0.002). Baseline RSES was associated with negative change in self-esteem (B = -0.22, p = 0.003). Taken together, these associations indicate that self-esteem tends to be stable over time, but those with the highest baseline scores do not retain as much of their self-esteem as those with lower baseline scores.

**Table 3:** Linear regression analysis of change in self-esteem at 9 months follow-up among control group participants with Type 1 DM 18–55 years of age (N = 83).

		Partly adjusted model ^a^^b^				Fully adjusted model ^c^			
		B	95% CI		P	B	95% CI		P
Self-esteem at baseline^d^		-0,17	-0.30	-0.05	0.006	-0.22	-0.37	-0.08	0.004
Sex		-1.24	-2.59	0.11	0.07	-2.16	-3.58	-0.74	0.004
Age		-0.01	-0.08	0.05	0.67	-0.01	-0.10	0.08	0.86
University education		0.40	-1.05	1.85	0.58	0.46	-0.97	1.88	0.52
Living alone		0.17	-1.62	1.96	0.85	1.02	-0.81	2.85	0.27
Employed		0.55	-0.37	1.47	0.24	0.38	-0.51	1.27	0.40
Long-term complications		1.85	0.47	3.23	0.009	2.76	1.32	4.21	<0.001
Pump		0.65	-0.73	2.02	0.35	0.61	-0.86	2.08	0.41
SMBG^e^									
	1–3 times per day	0.51	-0.11	2.13	0.53	0.52	-1.01	2.04	0.50
	Less than daily	0.05	-1.66	1.77	0.95	0.07	-1.64	1.78	0.94
Diabetes duration		-0.03	-0.09	0.04	0.40	-0.07	-0.15	0.02	0.11
Severe hypoglycemia		-1.66	-2.99	-0.32	0.02	-1.41	-2.77	-0.05	0.04
HbA_1c_ (%)		-0.24	-0.84	0.36	0.43	-0.37	-1.02	0.28	0.26
Autonomy support^f^		-0.03	-0.55	0.49	0.91	-0.18	-0.75	0.39	0.53
Autonomy-driven motivation^g^		0.18	-0.50	0.86	0.60	0.50	-0.48	-0.29	1.25
Self-management competence^h^		0.03	-0.48	0.55	0.90	0.01	-0.54	0.56	0.97
Overall R square		0.09^i^				0.43			
Change in R square						0.34			0.005

^a^Unstandardized regression coefficients

^b^Each cell in this column represents the coefficients from separate regression models for each predictor adjusted only for self-esteem at baseline

^c^Each cell in this column represents the coefficients from a multivariate regression analysis adjusted for self-esteem at baseline and all other predictor variables listed in the table

^d^RSES, Rosenberg Self-Esteem Scale

^e^Self-monitored blood glucose measurements, with ≥4 times per day as the reference category

^f^HCCQ, Health Care Climate Questionnaire

^g^TSRQ, Treatment Self-Regulation Questionnaire, Autonomy

^h^PCDS, Perceived Competence in Diabetes Scale

^i^R square from regression model with RSES at baseline as the only predictor variable

## Discussion

We identified relationships of perceived autonomy support, autonomy-driven motivation and self-perceived diabetes competence with self-esteem. Autonomy support was associated with higher autonomy-driven motivation, which in turn was associated with higher perceived diabetes competence, which in turn was associated with better self-esteem. Being female also predicted lower baseline self-esteem. We found that the strongest predictors of change in self-esteem at follow-up were long-term complications (specifically foot-related problems) and being female.

According to SDT, relatedness is defined as ‘a sense of affiliation with or belonging to others to whom one feels connected’ [31]. In the current study autonomy support from HCPs was found to be important driver of self-esteem by fostering autonomy-driven motivation and diabetes self-management competence. Connectedness to significant others was not measured directly, however, the variable ‘Living alone’ had a negative association with self-esteem. The association was not significant and should thus be interpreted with caution, but the negative association could indicate that living with a significant other is associated with better self-esteem. Spenceley et al. [32] found that assistance from spouse was helpful in maintaining expected social roles. Moreover, living alone in a Western culture is regarded as a stigma and can interfere with individuals’ self-esteem [33, 34]. Alternatively, support from a significant other may enhance patient self-management, thereby elevating self-esteem [35, 36].

In the previous intervention study from which the current data were obtained [23], we found that control group participants dropped in self-esteem at follow-up, whereas the intervention group remained stable (see Fig 1). A multi-centre intervention study examining the impact of a structured education program also found that self-esteem in the intervention group remained stable whereas self-esteem dropped in one of the control groups post intervention [37], but the authors were not able to determine why a drop in self-esteem occurred or who experienced a drop in self-esteem. Our study revealed that females had lower self-esteem at baseline and that being a female predicted a decrease in self-esteem at follow-up. These findings are congruent with a longitudinal cohort study among young adults with T1DM where women reported lower self-esteem than men [38] and the fact that women generally are found to have lower self-esteem than men [39].

Interpreting the somewhat unexpected positive association between change in self-esteem over time and long-term complications, especially foot-related problems, is challenging. Diabetic foot ulcers are serious complications [40], possibly causing devastating consequences like amputation [41] or death [42]. The risk of re-ulceration is 25–80% within a year [43, 44]. Emotional consequences of living with a diabetic foot ulcer are complex, with a feeling of powerlessness being prominent [45]. One possible explanation is that patients may have experienced healing of a diabetic foot ulcer as a consequence of a comprehensive and multidisciplinary treatment approach with frequent consultations until healing of the ulcer is a reality. A Norwegian study exploring the experiences of adults receiving treatment for diabetic foot ulcers found the most important elements to be competence of HCPs, continuity of care and easy access to healthcare services [46] which may have contributed to positive psychological outcomes and a sense of efficacy in dealing with the condition.

Self-esteem has been regarded as an essential part of building the individual’s personality, and personality has been regarded somewhat immutable [47]. At the same time, continuity or change of personality traits during a life course has been heavily discussed [48]. Thus, it is interesting that those with a higher level of self-esteem at baseline experienced a significant decrease in self-esteem at follow-up. This finding may reflect a regression to the mean as a result of measurement error or non-systematic fluctuations in measured levels of self-esteem. Understanding this pattern would require more frequent measures of self-esteem over a longer time period.

Some limitations of the present study should be acknowledged. First, the study’s generalizability may be restricted; although participants were randomly assigned to the control group that provided data for this study, the response rate for the parent study was only 37%, and we were not able to assess differences between participants and non-participants. Second, the study did not include objective measures of the participants’ ability to self-manage the condition, so it was not possible to determine whether the association between perceived self-management competence and self-esteem was mediated by quality of self-care. In addition, it would have been of interest to have number of SMBG as a continuous variable rather than in categories in order to study persons who measure very frequently more in detail. We did however try different combinations of categories and none showed any association with RSES. Also, the study did not include measures of change in factors that might have contributed to change in self-esteem, e.g., post-baseline episodes of severe hypoglycemia, HbA1_c_ levels, healing of foot problems, autonomy support/motivation or diabetes competence. Last, the analyses had an exploratory nature and several significance tests were done. We can thus not rule out the possibility that some of the significant findings were due to chance. We used 0.01 as significance level instead of 0.05 to reduce some of the risk of Type 1 error, but there could still be some Type I errors, especially for findings with p-values close to 0.01. We therefore encourage the reader to interpret the results with caution.

To summarize, the cross-sectional and longitudinal predictors of variations in self-esteem among adults with a suboptimally regulated T1DM have been evaluated within the conceptual framework of Self-Determination Theory. We found that perceived autonomy support, autonomy-driven motivation and diabetes competence were significant predictors of self-esteem in cross-sectional analysis, with the latter having the strongest relationship. Factors that interfere with the individual’s self-esteem over time included complications and female gender. Finally, we observed a tendency for self-esteem to manifest a regression to the mean, with those who had higher self-esteem at baseline experiencing a decline in self-esteem over time. Clinicians seeking to optimize psychological outcomes among their diabetes patients should consider the ways these factors contribute to self-esteem.

## Supporting information

S1 Data Dataset used in analyses, including explanation of variables. (XLS)
